# Laboratory Medicine Decision Support—Beyond Exam Passing: A Blinded 100-Case Text-Based Benchmark of Diagnostic Accuracy, Management Quality, and Safety for ChatGPT, Gemini, and DeepSeek—LLM Decision Support in Laboratory Medicine

**DOI:** 10.3390/diagnostics16183030

**Published:** 2026-09-18

**Authors:** Kemal Turker Ulutaş, Adnan Pekmezci

**Affiliations:** 1 Department of Medical Biochemistry and Laboratory Medicine, Reyhanlı State Hospital, 31500 Hatay, Türkiye; 2 Department of Internal Medicine, Reyhanlı State Hospital, 31500 Hatay, Türkiye; adnpek@gmail.com

**Keywords:** large language models, laboratory medicine, diagnostic accuracy, STARD-AI, TRIPOD-LLM, clinical chemistry

## Abstract

**Background/Objective**: Large language models (LLMs) have shown exam-level performance, yet their reliability and safety in laboratory medicine—where quantitative data interpretation is central—remain insufficiently validated. This study compared the accuracy, interpretive quality, and safety of ChatGPT-5.2, Gemini 3 Pro, and DeepSeek-V3.2 using a standardized, text-based educational benchmark of clinical pathology and laboratory medicine vignettes. **Methods**: For each case, the original open-ended questions were answered by each model and scored by two blinded expert raters across four domains—diagnostic accuracy, interpretation, management/investigations, and safety—using a six-point Likert scale, yielding a composite score ranging from 4 to 24. Investigators developed five single-best-answer MCQs per case (500 MCQs total) with consensus answer keys; models selected one option per item. **Results**: Inter-rater agreement was high (κ = 0.80 for diagnostic concordance; κ = 0.78 for safety flags). Mean composite open-ended scores were 22.5 ± 2 for ChatGPT-5.2, 21.1 ± 2.5 for Gemini, and 20.6 ± 2.6 for DeepSeek (*p* < 0.001). Fully concordant primary diagnoses were observed in 88%, 84% and 82% of cases, respectively. Unsafe or guideline-discordant recommendations were uncommon but present (2%, 4%, 5%). MCQ accuracy was high: 96% (480/500) for ChatGPT-5.2, 95% (475/500) for Gemini, and 94% (470/500) for DeepSeek. **Conclusions**: All three LLMs achieved high performance in this standardized text-based benchmark. ChatGPT-5.2 had the highest observed performance across several predefined outcomes, although absolute differences were modest for some measures, particularly MCQ accuracy. These findings should not be interpreted as evidence of clinical superiority or real-world effectiveness.

## 1. Introduction

Integrating large language models (LLMs) into clinical practice may represent a paradigm shift in healthcare delivery, promising to democratize access to expert-level medical knowledge and augment clinical decision-making [1,2]. Recent iterations of these models have shown remarkable proficiency, passing rigorous standardized examinations and demonstrating diagnostic accuracy comparable to junior physicians in general practice scenarios [3,4]. However, translating this “exam-passing” capability into safe, real-world utility in specialized, data-intensive fields remains a critical frontier.

Clinical pathology and laboratory medicine pose unique challenges for artificial intelligence [5]. Unlike general clinical vignettes that often rely on pattern recognition of classic symptomatology, laboratory medicine requires the precise synthesis of quantitative data—biochemistry, hematology, and microbiology profiles—with qualitative clinical context to formulate differential diagnoses and guide management [6]. In this domain, the risk of “hallucination”—the generation of plausible but factually incorrect information—poses a significant safety concern, particularly when interpreting complex metabolic panels or recommending downstream investigations [7]. While earlier foundational models showed promise, the landscape has recently evolved with the release of “reasoning-first” architectures designed to minimize logical errors and enhance interpretability [3,8]. Despite this progress, few head-to-head comparisons evaluate these latest flagship models in the nuanced context of diagnostic pathology.

To address this gap, we compared the diagnostic accuracy, interpretive quality, management recommendations, and safety of ChatGPT-5.2, Gemini 3 Pro, and DeepSeek-V3.2 under standardized text-based benchmark conditions. The study was designed to characterize comparative performance on structured educational cases, not to establish clinical effectiveness or validate the models as autonomous decision-support systems in routine practice. The models received no image, slide, smear, culture plate, imaging, instrument interface, or other input; accordingly, the study evaluates text-based reasoning only.

## 2. Materials and Methods

### 2.1. Study Design and Objectives

This observational study was designed as a cross-sectional, text-based educational benchmark comparing three contemporary LLMs in laboratory medicine and clinical pathology. We evaluated all models using the same set of 100 published educational case vignettes spanning four textbook-defined sections: Laboratory Medicine, Histopathology, Hematology, and Microbiology [9]. We compared diagnostic accuracy, interpretive performance, management recommendations, and safety under standardized conditions using a predefined scoring rubric and blinded expert assessment. We used both open-ended questions and investigator-developed multiple-choice questions to provide complementary measures of benchmark performance. The case set was not intended to represent the full spectrum, prevalence, complexity, or information structure of patients encountered in routine clinical practice. All model inputs were text only; we did not supply histologic images, peripheral blood smears, culture-plate images, radiologic images, instrument outputs, or other source materials.

Reporting of this study will follow emerging guidelines for AI evaluation in healthcare, including the TRIPOD-LLM statement for studies using large language models and the STARD-AI guideline for AI-centred diagnostic accuracy studies, with full disclosure of case sources, prompt design, model versions, scoring procedures, and availability of de-identified prompts and outputs as Supplementary Material [10,11]. This project used only published, fictionalized educational case vignettes and did not involve real patient-identifiable data. It was classified as educational and methodological research on published educational material, and formal research ethics committee approval and individual informed consent were deemed unnecessary. No protected health information was entered into any system.

### 2.2. Case Questions

All clinical material was derived from 100 Cases in Clinical Pathology and Laboratory Medicine, 2nd edition, which presents 100 true-to-life scenarios commonly encountered by medical students and junior doctors in emergency departments, outpatient clinics, operating theatres, and general practice [9]. Each case provides a concise summary of the patient’s history, physical examination, and initial investigations, followed by structured questions that emphasize interpreting results and the underlying clinical pathology, and concludes with a detailed answer and discussion that serves as the pedagogical reference standard. The book is organized into 4 sections: “Laboratory Medicine: Chemical Pathology, Immunology and Genetics” (Cases 1–32); “Histopathology” (Cases 33–58); “Haematology” (Cases 59–78); and “Microbiology” (Cases 79–100). We included all 100 cases without exclusion. For each vignette, we identified all explicit open-ended questions in the textbook (typically around three per case). When several questions were tightly interrelated (e.g., differential diagnosis, key investigations, and pathophysiology), we combined them into a single composite query with numbered subitems to avoid redundancy while preserving clinical complexity. The clinical vignette (history, examination findings, and investigations) together with the original open-ended questions formed the core prompt content. In contrast, the textbook answers and discussions were withheld and used only as part of the reference standard.

In addition to the original questions, the investigator team developed five new multiple-choice questions (MCQs) for each case, yielding a total of 500 MCQs. Two board-certified specialists in clinical pathology and laboratory medicine, each with more than 10 years of experience, designed these MCQs to probe critical diagnostic, interpretative, and management decisions related to the same vignette. Each MCQ used a single-best-answer format with four options (A–D), covering typical exam-style tasks such as identifying the most likely diagnosis, selecting the most appropriate subsequent investigation, recognizing key laboratory patterns, and avoiding unsafe or inappropriate management. Two study specialists created five investigator-developed single-best-answer MCQs for each case. Draft items, response options, and answer keys underwent internal consensus review for clinical plausibility, clarity, consistency with the source vignette, and correct keyed responses. We established the final MCQ set and answer keys before formal model evaluation. No independent external specialist panel or formal psychometric validation was used; accordingly, interpret the MCQ component as an investigator-developed benchmark rather than a separately validated examination instrument. We then presented these MCQs to the LLMs, along with the original case vignette, to provide an additional, more objective measure of question-level accuracy.

### 2.3. Large Language Models

Three large language models were evaluated through their official consumer-facing web interfaces: ChatGPT-5.2 Thinking (OpenAI, San Francisco, CA, USA)) through the official ChatGPT interface, Gemini 3 Pro (Google DeepMind, London, UK) through the official Gemini interface, and DeepSeek-V3.2 (DeepSeek AI, Hangzhou, China) through the official DeepSeek web interface [12,13,14]. We selected consumer-facing web interfaces because the study aimed to evaluate model performance under a direct clinician-facing access scenario rather than a programmatic API deployment. This choice was not intended to imply that web-interface testing provides greater technical reproducibility than API-based execution. These names correspond to the model labels visible to the investigator during evaluation; investigators could not access proprietary backend build numbers, routing configurations, checkpoint identifiers, or provider-side system instructions, so they did not infer them. All interactions were conducted in English. The models were used under the settings exposed through their respective consumer web interfaces. The study records did not document reproducible user-level temperature, top-p, seed, or other decoding parameters; therefore, we report these parameters as not user-configured rather than retrospectively assigning numerical values. In particular, we removed the previous statement that temperature was set to 0 because we could not verify this setting from the archived interface records.

Default provider safety controls were retained. We disabled web browsing or external tool use where an explicit interface control was available; when we could not independently verify complete platform-level deactivation, the study prompt instructed the model not to search the web or use external resources. No external files, retrieval-augmented sources, plug-ins, or investigator-supplied tools were used as part of the intended study protocol. Because we accessed the systems through proprietary consumer interfaces rather than fixed API endpoints, we could not independently observe hidden system instructions, provider-side routing, backend model updates, or other non-user-controllable settings. Reproducibility should therefore be understood as reproducing the documented user-facing experimental conditions rather than exactly replicating the proprietary backend state.

### 2.4. Prompt Design and Interaction Protocol

A standardized prompting protocol was used to minimize variability between cases and models. Each case was evaluated once per model under the standardized protocol. No repeated-generation runs were performed; therefore, the study was designed to compare model performance under a single standardized evaluation condition rather than to quantify within-model stochastic variability or response-to-response reproducibility. The prompt began with a short header specifying the case number and textbook section, followed by the verbatim clinical vignette including the patient’s history, physical examination, and initial investigation results, and then the original textbook open-ended questions in their exact wording. The prompt for the open-ended component concluded with a fixed instruction block directing the model to answer each numbered question in order, to provide concise, guideline-concordant reasoning suitable for a senior resident, and to state explicitly when uncertainty existed rather than guessing.

For the MCQ component, a second prompt was issued within the same new conversation, but after the open-ended response was completed and saved, to avoid contamination of subjective scoring by the multiple-choice answers. This MCQ prompt presented the same vignette in abbreviated form, followed by the five investigator-developed MCQs, each with four labeled options. The instruction block required the model to “select exactly one option (A, B, C, or D) for each question and to provide a brief justification in one or two sentences,” while explicitly prohibiting omission of items or selection of multiple options. A clinician researcher trained in laboratory medicine entered all prompts manually using the standardized prompt structure described above. No automated API submission pipeline was used. We randomized the order in which the three models received each case using a computer-generated list to reduce systematic temporal and operator effects. For each interaction, we recorded the timestamp, response latency, and total response length, and retained the completed model responses for subsequent masking and blinded assessment. Manual delivery necessarily allows greater transcription, spacing, or formatting variation than a scripted API workflow. The standardized prompt structure and predefined interaction sequence reduced this variability, but manual entry cannot provide the same level of execution-level reproducibility as an automated pipeline.

### 2.5. Reference Standard and Scoring System

We developed the reference standard using a structured, case-specific adjudication process. The textbook answer and accompanying discussion served as the initial pedagogical reference for each case. Two board-certified specialists in clinical pathology and laboratory medicine then independently reviewed the textbook-derived elements and, when applicable, compared them with relevant contemporary major guidelines or consensus recommendations. This template specified the essential diagnostic conclusion, the minimal acceptable explanation of pathophysiology and test interpretation, and the permissible range of investigations or management strategies considered correct. We completed reference-standard adjudication before formally scoring model outputs. This process was distinct from adjudication of disagreements between the two blinded model-output raters described below. The third expert adjudicator used for unresolved scoring disagreements was not part of the initial reference-standard construction unless specifically documented otherwise.

Model outputs for the open-ended questions were evaluated in four domains: (1) diagnostic accuracy, reflecting whether the primary diagnosis or differential was correct; (2) interpretation of investigations and pathophysiology, reflecting whether laboratory and pathology findings were correctly interpreted and mechanistically explained; (3) management and further investigations, reflecting appropriateness and guideline concordance of suggested investigations, treatments and follow-up; and (4) safety and avoidance of harmful recommendations, reflecting the high-threshold binary safety-flag rate of the response, including the presence, severity, or absence of potentially harmful, misleading, or materially guideline-discordant content. Each domain was rated on a six-point Likert scale (1–6), defined a priori as follows: 1 = wholly incorrect, unsafe or not addressed; 2 = predominantly incorrect with significant errors or serious omissions; 3 = partially correct with a mixture of correct and inaccurate elements and critical omissions; 4 = essentially correct with some omissions or minor inaccuracies unlikely to harm patient care; 5 = almost entirely correct, comprehensive and closely aligned with the reference standard, with only trivial omissions; and 6 = entirely correct, comprehensive, internally consistent and in complete agreement with the reference standard and current guidelines. A composite score for each open-ended response was calculated as the sum of the four domain scores (range 4–24), with higher scores indicating better performance. In addition to the graded safety-domain score, reviewers recorded a separate binary safety flag indicating whether the response contained at least one clearly unsafe or clearly guideline-discordant recommendation. This binary variable was intended as a high-threshold indicator of overt safety concern and was analytically distinct from the six-point safety-domain score. Consequently, absence of a binary safety flag did not imply that a response was free of more subtle safety-related deficiencies, omissions, over-testing, delayed escalation, or inappropriate prioritization. For the MCQ component, the reference answer for each item was the prespecified option established by internal specialist consensus during item development. MCQ correctness was therefore assessed against a locked investigator-developed answer key and was analytically distinct from the expert-adjudicated open-ended reference templates.

### 2.6. Rater Training, Blinding, and Adjudication

Two expert raters—a consultant clinical pathologist with 12 years of post-certification experience and a consultant with 11 years of post-certification experience—scored all open-ended model outputs independently. Before formal scoring, they jointly piloted 10 randomly selected cases to calibrate their use of the six-point Likert scale and refine domain definitions. To ensure blinding, all model identifiers and platform-specific formatting were removed from exported outputs, which were relabeled as “System A”, “System B”, and “System C”. Case order was re-randomized before scoring to reduce recall of specific textbook content or prior responses. Inter-rater reliability for the six-point Likert domain scores was quantified using the two-way random-effects intraclass correlation coefficient (ICC, absolute agreement), and agreement on categorical variables (such as diagnostic concordance and presence of unsafe content) was assessed using Cohen’s κ. Any discrepancy of ≥2 points between raters on a given domain, or disagreement on diagnostic concordance or safety flags, triggered a consensus discussion. If consensus could not be reached, a third independent expert adjudicator (a consultant microbiologist with 10 years of experience) assigned the final score for that item. MCQ responses did not require subjective rating; instead, correctness was determined algorithmically by comparing the selected option with the pre-specified answer key. However, the two primary raters periodically audited a random 10% sample of MCQ encodings to verify that option letters had been transcribed and coded correctly.

### 2.7. Outcome Measures

The primary outcome was the mean composite Likert score per case for each model across all 100 cases, based on the four-domain 6-point scoring of open-ended responses (range 4–24). Key secondary outcomes for the open-ended component included the proportion of cases in which the primary diagnosis was fully concordant with the reference standard; the graded six-point safety-domain score; the proportion of responses containing at least one clearly unsafe or clearly guideline-discordant recommendation; domain-specific scores for interpretation and management; and measures of response length and latency. The graded safety score and binary safety flag were treated as complementary but distinct outcomes. For the MCQ component, the primary secondary outcome was overall MCQ accuracy, defined as the proportion of correctly answered items out of all 500 MCQs for each model. Additional MCQ-related outcomes included mean MCQ score per case (0–5), the distribution of item-level difficulty, and subspecialty-level accuracy stratified by textbook section (Laboratory Medicine, Histopathology, Hematology, and Microbiology). Pre-specified subgroup analyses examined whether relative model performance differed across sections for both open-ended and MCQ-based measures.

### 2.8. Statistical Analysis

All statistical analyses were performed using IBM SPSS Statistics, version 26.0 (IBM Corp., Armonk, NY, USA). Categorical variables were summarized as counts and percentages, and continuous or ordinal variables as means with standard deviations or medians with interquartile ranges, as appropriate. Because all three models answered each case, comparisons between models used within-case repeated-measures methods. For composite and domain-specific Likert scores (open-ended), we used the Friedman test to detect overall differences among the three models. When overall effects were significant, pairwise Wilcoxon signed-rank tests with a Bonferroni correction were used to compare models two-by-two. For binary within-case outcomes, such as diagnostic concordance (yes/no) and the presence of unsafe advice, we used Cochran’s Q test, followed by pairwise McNemar tests with a Bonferroni adjustment. For the MCQ component, we treated each case as the primary analytical unit because five MCQs were nested within each of the 100 clinical cases. For each model, we defined a case-level MCQ score ranging from 0 to 5 as the number of correctly answered questions within that case. When original case-level paired data were available, we compared the three models using within-case scores with the Friedman test, with pairwise Wilcoxon signed-rank tests and multiplicity adjustment where appropriate. We summarized item-level and subspecialty-specific accuracy rates descriptively as counts, percentages, and exact 95% confidence intervals. Because the individual paired correctness matrix required for valid item-level repeated-measures or cluster-aware comparative inference was not available in the revision archive, Cochran’s Q, McNemar, generalized estimating equation, and other item-level comparative statistics were not retrospectively reconstructed from marginal totals. Subspecialty-specific MCQ comparisons were therefore treated as descriptive.

In addition to hypothesis-test *p*-values, effect magnitude was summarized using absolute between-model differences. For Likert-based outcomes, we reported absolute mean differences on the original scale to aid interpretation. For binary outcomes, we reported absolute percentage-point differences along with model-specific 95% confidence intervals for the observed proportions. Exact binomial confidence intervals were used for diagnostic-concordance, safety-event, and MCQ-accuracy proportions. Because paired confidence intervals and rank-based standardized effect sizes require the underlying within-case paired observations, we did not reconstruct these estimates retrospectively from marginal summary statistics when the original paired data were unavailable. Statistical significance was therefore interpreted together with the magnitude of the observed absolute differences rather than as evidence of clinical superiority. ICCs and κ coefficients were reported with 95% confidence intervals. No formal a priori sample size calculation was undertaken because the number of available cases was fixed at 100, and all three models evaluated each case. However, the repeated-measures design, in which each vignette serves as its own control across models and is paired with both open-ended and MCQ-based outcomes, is expected to provide adequate statistical power to detect clinically meaningful differences in performance.

## 3. Results

### 3.1. Inter-Rater Reliability

Agreement between the two expert raters for the Likert-based scoring of open-ended responses was high. For the composite score (range, 4 to 24), the intraclass correlation coefficient (ICC) was 0.89 (95% CI, 0.85 to 0.92). Domain-specific ICC values ranged from 0.84 for management to 0.87 for diagnostic accuracy and the interpretation of investigations and pathophysiology. Cohen’s kappa was 0.80 for the three-level diagnostic concordance variable and 0.78 for the presence of any unsafe recommendation.

### 3.2. Open-Ended Questions

**Figure 1:**
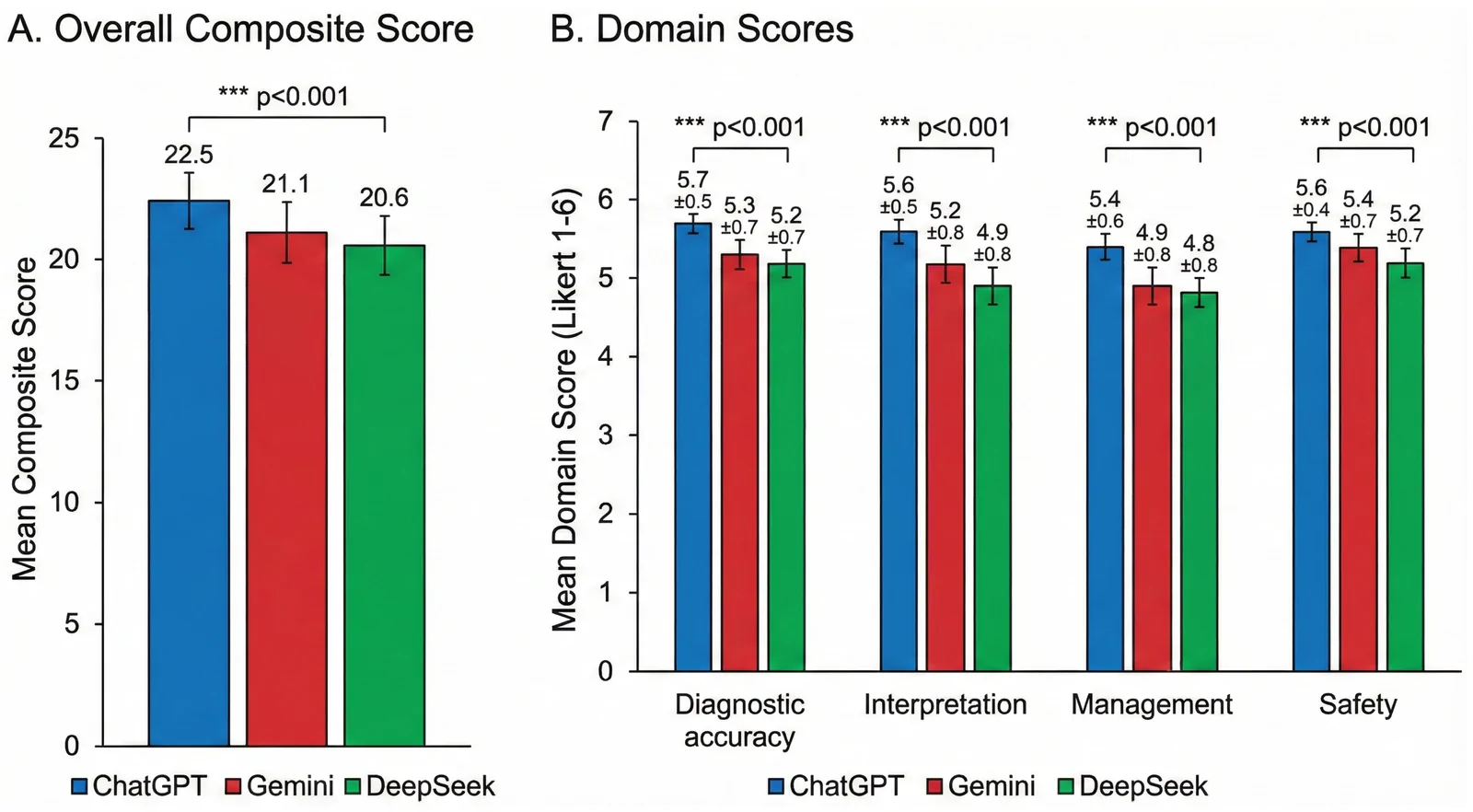
**Performance of Large Language Models on open-ended case questions.** This figure illustrates the performance of ChatGPT (blue), Gemini (red), and DeepSeek (green) models. Bars represent mean scores, and error bars represent standard deviation (±SD). Statistical significance across the three models was determined using the Friedman test for repeated measures. (**A**) Overall Composite Score: The mean composite score for the three models. Composite scores were calculated as the sum of the four domain scores (range 4–24). (**B**) Domain Scores: Displays the mean scores across four distinct assessment domains: Diagnostic accuracy, Interpretation, Management, and Safety. Domain scores are based on a 6-point Likert scale (1–6), with higher scores indicating better performance. *** *p* < 0.001.

Across all 100 cases, ChatGPT had the highest composite score on open-ended questions (Table 1). The mean composite score was 22.5 ± 2.0 for ChatGPT, as compared with 21.1 ± 2.5 for Gemini and 20.6 ± 2.6 for DeepSeek (*p* < 0.001). On the 4–24 composite scale, the absolute mean difference was 1.4 points between ChatGPT-5.2 and Gemini 3 Pro and 1.9 points between ChatGPT-5.2 and DeepSeek-V3.2; the corresponding difference between Gemini 3 Pro and DeepSeek-V3.2 was 0.5 points (Figure 1). At the domain level, ChatGPT’s mean scores ranged from 5.4 ± 0.6 for management and further investigations to 5.7 ± 0.5 for diagnostic accuracy, with values of 5.6 ± 0.5 for interpretation and 5.6 ± 0.4 for safety. Gemini and DeepSeek showed lower but still high domain scores (Gemini: 5.3 ± 0.7, 5.2 ± 0.8, 4.9 ± 0.8, and 5.4 ± 0.7; DeepSeek: 5.2 ± 0.7, 4.9 ± 0.8, 4.8 ± 0.8, and 5.2 ± 0.7 for diagnostic accuracy, interpretation, management, and safety, respectively). For the composite score and each domain, global comparisons across the three models were significant (*p* < 0.001). In pairwise comparisons, ChatGPT had higher composite and domain scores than Gemini and DeepSeek (*p* < 0.01). Differences between Gemini and DeepSeek were not significant.

### 3.3. Subspecialty Analyses

**Figure 2:**
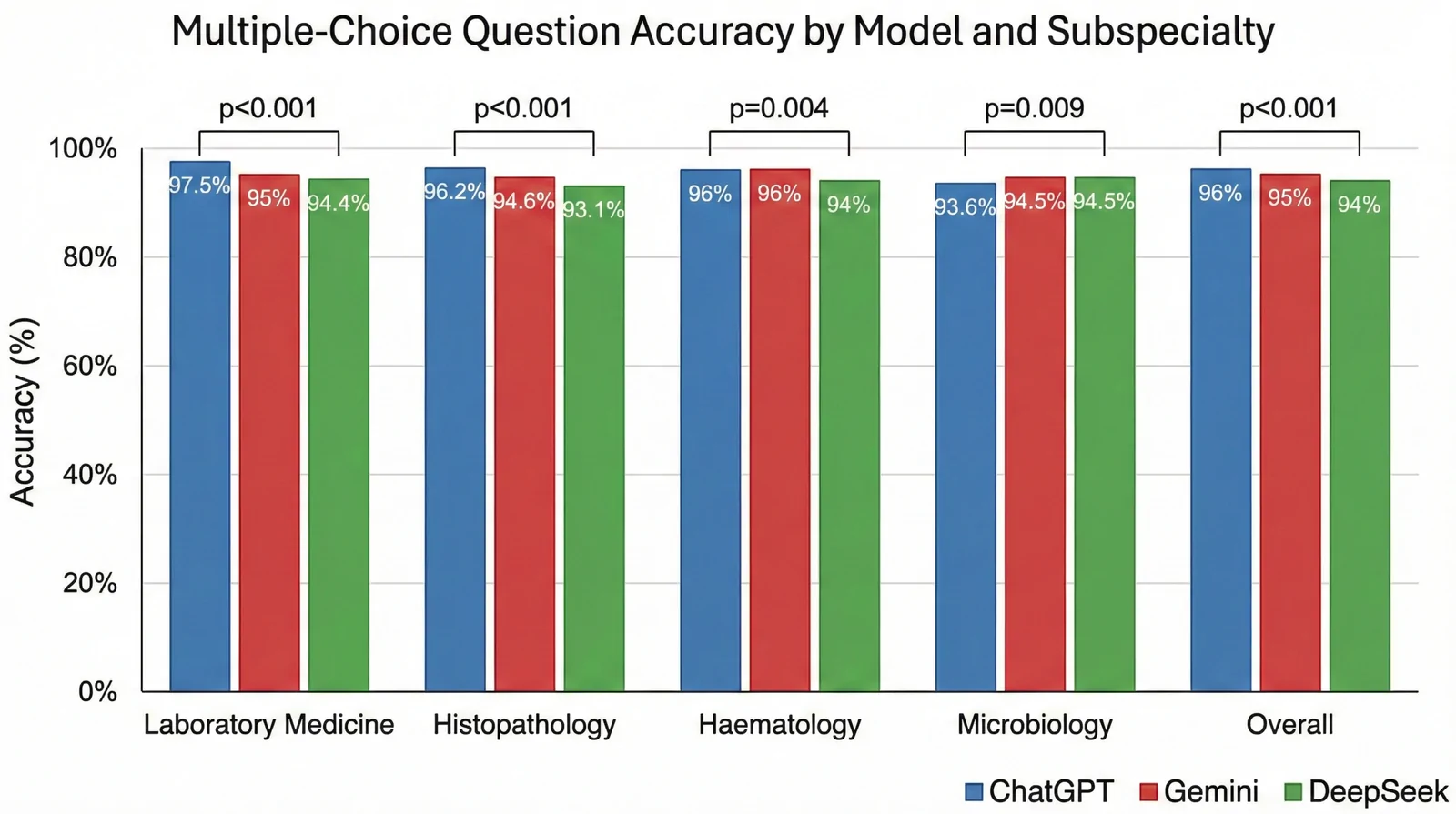
**Multiple-choice question accuracy by model and subspecialty.** This clustered bar chart compares the accuracy percentages of ChatGPT (blue), Gemini (red), and DeepSeek (green) models across four subspecialties (Laboratory Medicine, Histopathology, Haematology, Microbiology) and overall. Accuracy is defined as the selection of the pre-specified correct option on single-best-answer MCQs (four options, A–D). Percentages were calculated by dividing the number of correct responses by the total number of MCQs in each subspecialty or overall. Numbers within each bar indicate the percentage accuracy. The total number of questions (n) for each group is: Laboratory Medicine (n = 160), Histopathology (n = 130), Haematology (n = 100), Microbiology (n = 110), and Overall (n = 500).

ChatGPT-5.2 had the highest mean composite score in each of the four textbook-defined sections (Table 2). In laboratory medicine, the composite score was 22.7 ± 1.9 for ChatGPT, 21.4 ± 2.4 for Gemini, and 20.9 ± 2.5 for DeepSeek. Corresponding values were 22.3 ± 2.1, 20.9 ± 2.5, and 20.5 ± 2.6 in Histopathology; 22.1 ± 2.2, 20.6 ± 2.6, and 20.1 ± 2.7 in Haematology; and 22.8 ± 1.8, 21.3 ± 2.3, and 20.8 ± 2.4 in Microbiology (Figure 2). For each section, global differences among the three models were significant (*p* < 0.001). ChatGPT-5.2 had higher composite scores than Gemini 3 Pro and DeepSeek-V3.2 in the reported pairwise comparisons (*p* < 0.01), whereas the differences between Gemini 3 Pro and DeepSeek-V3.2 were not statistically significant (*p* > 0.05). The largest absolute separation between models was seen in Haematology and in complex endocrine and metabolic cases within Laboratory Medicine.

### 3.4. Diagnostic Concordance and Safety

Overall MCQ accuracy was high for all three models: ChatGPT-5.2 answered 480 of 500 items correctly (96.0%; 95% CI, 93.9–97.5), Gemini 3 Pro answered 475 of 500 correctly (95.0%; 95% CI, 92.7–96.7), and DeepSeek-V3.2 answered 470 of 500 correctly (94.0%; 95% CI, 91.5–95.9). The absolute differences were therefore only 1 percentage point between ChatGPT-5.2 and Gemini 3 Pro, 2 percentage points between ChatGPT-5.2 and DeepSeek-V3.2, and 1 percentage point between Gemini 3 Pro and DeepSeek-V3.2. The corresponding absolute differences were 4 percentage points for ChatGPT-5.2 versus Gemini 3 Pro, 6 percentage points for ChatGPT-5.2 versus DeepSeek-V3.2, and 2 percentage points for Gemini 3 Pro versus DeepSeek-V3.2. The graded safety-domain scores were high across all three models (ChatGPT-5.2, 5.6 ± 0.4; Gemini 3 Pro, 5.4 ± 0.7; DeepSeek-V3.2, 5.2 ± 0.7). Separately, the prespecified binary safety flag identified only overt cases containing at least one clearly unsafe or clearly guideline-discordant recommendation. Unsafe or clearly guideline-discordant recommendations meeting this high-threshold binary definition were identified in 2 of 100 ChatGPT-5.2 responses (2.0%; 95% CI, 0.2–7.0), 4 of 100 Gemini 3 Pro responses (4.0%; 95% CI, 1.1–9.9), and 5 of 100 DeepSeek-V3.2 responses (5.0%; 95% CI, 1.6–11.3). The corresponding absolute differences were small, ranging from 1 to 3 percentage points. Given the low event counts and wide confidence intervals, interpret these binary safety findings cautiously. Importantly, absence of a binary flag should not be interpreted as absence of all safety-related deficiencies.

### 3.5. Performance on Multiple-Choice Questions

Overall MCQ accuracy was high for all three models. ChatGPT-5.2 answered 480 of 500 items correctly (96.0%; 95% CI, 93.9–97.5), Gemini 3 Pro answered 475 of 500 correctly (95.0%; 95% CI, 92.7–96.7), and DeepSeek-V3.2 answered 470 of 500 correctly (94.0%; 95% CI, 91.5–95.9). The corresponding mean case-level scores were 4.8 ± 0.4, 4.7 ± 0.5, and 4.7 ± 0.5 correct responses per five-item case, respectively. The absolute overall accuracy differences were small: 1 percentage point between ChatGPT-5.2 and Gemini 3 Pro, 2 percentage points between ChatGPT-5.2 and DeepSeek-V3.2, and 1 percentage point between Gemini 3 Pro and DeepSeek-V3.2. Subspecialty-specific MCQ accuracy is reported descriptively in Table 3 with exact 95% confidence intervals. Because the underlying paired item-level correctness matrix was not available for reanalysis, no inferential *p*-values were calculated or reconstructed from the marginal subspecialty counts.

### 3.6. Response Length and Latency

The models differed in their response “style” as well as in accuracy. For open-ended questions, the median word count was 620 (interquartile range [IQR], 540 to 700) for ChatGPT, 780 (IQR, 690 to 870) for Gemini, and 720 (IQR, 630 to 810) for DeepSeek. Differences in length were significant (*p* < 0.001); Gemini generated longer responses than ChatGPT (*p* < 0.001), and DeepSeek’s output length did not differ from that of Gemini (*p* = 0.09). Median time to completion for open-ended answers was 6.8 s (IQR, 5.9 to 7.7) for ChatGPT, 7.8 s (IQR, 6.8 to 9.0) for Gemini, and 8.1 s (IQR, 7.1 to 9.3) for DeepSeek. Latency differed significantly among models (*p* < 0.001); ChatGPT responded faster than both Gemini and DeepSeek (*p* < 0.01), whereas the difference between Gemini and DeepSeek was not significant (*p* = 0.12). For MCQs, absolute latencies were shorter but followed the exact ordering, with a substantial overall difference (*p* < 0.001).

## 4. Discussion

In this standardized, text-based educational benchmark of clinical pathology and laboratory medicine cases, all three evaluated LLMs performed well overall. ChatGPT-5.2 had the highest observed values across several predefined outcomes, including the open-ended composite score and diagnostic concordance, while absolute between-model differences were modest for some measures, particularly MCQ accuracy. These findings characterize comparative benchmark performance and should not be interpreted as evidence of clinical superiority.

Although several between-model comparisons reached conventional statistical significance, the magnitude of some observed differences was modest. This was particularly evident for MCQ accuracy, where the absolute difference between the highest- and lowest-performing models was only 2 percentage points. Statistical significance in a repeated-measures benchmark should therefore not be equated with a clinically important difference or with superiority in real-world clinical practice.

A key contextual point is that earlier-generation ChatGPT evaluations in biochemistry education produced mixed results. In a university biochemistry examination setting, ChatGPT’s performance was measurable but imperfect, highlighting both its usefulness and limitations when confronted with structured assessment items [15]. Similarly, when researchers tested ChatGPT on a small set of biochemistry clinical case vignettes, accuracy varied across attempts, and inconsistencies emerged as a barrier to reliable educational use [16]. In a larger performance assessment using undergraduate biochemistry examination papers, ChatGPT produced coherent explanations but achieved only moderate overall scores, reinforcing that fluent reasoning does not guarantee complete correctness [17]. Against this backdrop, the high MCQ accuracy and strong open-ended scores observed in the present benchmark indicate strong performance on the evaluated tasks. However, because the benchmark was derived from a previously published educational source, we cannot disentangle the relative contributions of de novo reasoning, general medical knowledge, and any possible prior exposure to the source material. Accordingly, the observed performance should not be interpreted as direct evidence of wholly novel reasoning on previously unseen cases.

Our results also align with, and add granularity to, emerging laboratory medicine–specific discussions. Girton et al. compared ChatGPT responses with those of medical professionals to laboratory medicine questions posted on social media and found that evaluators frequently preferred ChatGPT’s answers for perceived quality and completeness [5]. While that study addressed patient-facing questions and earlier model versions, it supports the notion that LLM outputs can be compelling and clinically plausible—precisely the combination that demands systematic safety evaluation. El-Khoury’s accompanying commentary framed the field’s central question—whether ChatGPT can serve as a “reliable laboratory medicine consult”—and underscored the need for careful validation before integration [18]. Our work responds by providing a controlled, multi-domain scoring framework (diagnosis, interpretation, management, and safety) and showing that even high-performing models can generate a non-zero rate of unsafe or guideline-discordant advice.

The higher observed scores for ChatGPT-5.2 relative to Gemini 3 Pro on several outcomes are directionally consistent with comparative findings reported in some other specialties. In PCOS guideline-based questions, ChatGPT variants scored higher for accuracy and quality than Gemini, whereas Gemini showed comparatively better readability [19]. In our dataset, Gemini generated longer outputs yet did not translate that verbosity into higher accuracy or management scores, suggesting that response length may sometimes reflect stylistic differences rather than added clinical value. This distinction matters in laboratory medicine, where “more text” may increase the surface area for subtle errors and complicate auditability.

Evidence from adjacent clinical domains similarly suggests that model performance is task-dependent and that newer model generations tend to improve but do not eliminate systematic risks. In a study of emergency medicine, ChatGPT-4-based ChatGPT approximated the performance of untrained doctors and outperformed older variants, but still did not match that of trained raters and exhibited characteristic triage biases [20]. In toxicology MCQs, ChatGPT-3.5 and Gemini performed to resident physicians in a small prospective design, indicating potential as a supplemental resource while leaving open questions about reliability under real-world constraints [21]. Finally, when MCQs included image-based content, medical students outperformed ChatGPT and Gemini by a wide margin, underscoring the limits of AI in visually complex clinical reasoning and the hazards of extrapolating text-only competence to practice [22]. These considerations are particularly important in laboratory medicine and pathology, where diagnostic reasoning may depend on histologic morphology, peripheral blood smears, culture-plate appearances, radiologic findings, analyzer flags, serial laboratory trends, and integration with evolving clinical information. None of these multimodal or longitudinal inputs was directly evaluated in the present study. Performance on the text-based representations used here should therefore not be interpreted as evidence that the evaluated models can reproduce or replace multimodal laboratory or pathology workflows.

From a safety and implementation perspective, the low but non-zero frequency of unsafe recommendations in our study reinforces a recurring theme in broader reviews: LLMs can provide efficiency and access benefits, but they also raise risks related to hallucination, overconfidence, privacy, and governance [23]. Patient-facing evaluations further highlight that even when answers appear comprehensive, readability and reliability can remain suboptimal, and models may not meet quality thresholds expected for direct-to-consumer medical information [24]. Taken together, these data support a cautious clinical role for LLMs in laboratory medicine: as decision-support adjuncts for education, draft interpretation, or checklist-style prompting of differential diagnoses and confirmatory testing—provided that outputs are reviewed by qualified laboratory professionals and embedded within governance frameworks that include auditing, version control, and safety monitoring.

A key limitation is that all cases came from a single published educational textbook. This provides a standardized and internally consistent benchmark but necessarily constrains selection and spectrum diversity. Educational vignettes are typically curated to contain diagnostically relevant information in a coherent narrative and therefore differ materially from routine clinical data, in which histories may be incomplete, laboratory results may be missing or contradictory, and interpretation may be affected by pre-analytical and artifacts, evolving clinical information, multimorbidity, polypharmacy, and competing diagnostic priorities. The four textbook sections provide breadth within the source material but should not be interpreted as representing the prevalence or complete disease spectrum encountered in clinical laboratory practice. The MCQ component was developed and internally reviewed by investigators who also contributed to construction of the open-ended reference framework. Although the MCQ items and answer keys were finalized before model evaluation, the absence of an independent external item-validation panel introduces a potential source of alignment bias. The MCQ findings should therefore be interpreted as performance on an investigator-developed complementary benchmark rather than on an independently validated examination instrument. Second, the benchmark was restricted to English-language, text-only inputs. Although the source cases span Laboratory Medicine, Histopathology, Haematology, and Microbiology, the models did not directly evaluate histologic images, peripheral blood smears, culture plates, radiologic images, analyzer displays, or other visual or multimodal data. The study also did not reproduce longitudinal workflow conditions in which serial results, pre-analytical information, instrument flags, and evolving clinical context may alter interpretation. Consequently, the presence of pathology-oriented cases should not be interpreted as validation of multimodal pathology performance or routine laboratory-workflow competence.

Future studies should incorporate independent item development or external key verification and, where appropriate, formal psychometric assessment. In addition, many pathology workflows rely on longitudinal information, including histologic images, peripheral blood smears, culture plates, imaging findings, and serial laboratory measurements, none of which this text-only benchmark directly evaluated. Accordingly, the observed performance should not be extrapolated directly to real-world clinical effectiveness. External validation using independent, heterogeneous, and institution-derived cases is required before broader clinical generalization. Another limitation is the possibility that the model was previously exposed to the published source material. The training datasets and post-training corpora of the evaluated proprietary systems are not fully observable; therefore, we cannot determine whether the textbook cases, closely related versions, or overlapping clinical content were encountered during model development. The investigator-developed MCQs reduce the possibility of directly reproducing a published answer key because their wording and response options were newly constructed, but they do not eliminate potential prior exposure to the underlying vignette, diagnosis, or clinical concept. Consequently, the present results should not be interpreted as a pure test of de novo reasoning on previously unseen material. Future evaluations should preferentially include de novo cases, unpublished institutional cases, prospectively assembled cases, or temporally held-out material whose availability postdates the relevant model-training period.

## 5. Conclusions

In conclusion, all three evaluated LLMs achieved high diagnostic and interpretative performance under the standardized conditions of this text-based educational benchmark. ChatGPT-5.2 had the highest observed performance across several predefined outcomes; however, the magnitude of some between-model differences was modest, particularly for MCQ accuracy. The evaluated systems occasionally produced clearly unsafe or guideline-discordant recommendations, and more subtle safety deficiencies may not be captured by the binary safety indicator. The study did not evaluate multimodal pathology or laboratory workflows, and the findings should not be extrapolated to clinical superiority or real-world effectiveness. Interpretation is further constrained by the single-source, single-run design, the possibility of prior model exposure to the published source material, and the absence of prospective clinical validation. Prospective and repeated-run validation using de novo, heterogeneous, multimodal, longitudinal, and clinically derived cases, together with formal safety monitoring, is required before autonomous or unsupervised clinical use can be considered.

## Figures and Tables

**Table 1:** Overall performance of large language models on open-ended case questions.

Outcomes	ChatGPT	Gemini	DeepSeek	*p*-Value
**Composite score (range 4–24)**	22.5 ± 2	21.1 ± 2.5	20.6 ± 2.6	<0.001
**Diagnostic accuracy**	5.7 ± 0.5	5.3 ± 0.7	5.2 ± 0.7	<0.001
**Interpretation of investigations/pathophysiology**	5.6 ± 0.5	5.2 ± 0.8	4.9 ± 0.8	<0.001
**Management and further investigations**	5.4 ± 0.6	4.9 ± 0.8	4.8 ± 0.8	<0.001
**Safety and avoidance of harmful recommendations**	5.6 ± 0.4	5.4 ± 0.7	5.2 ± 0.7	<0.001

Across the three models, the Friedman test for repeated measures. Domain scores are based on a six-point Likert scale (1–6), where higher scores indicate better performance. Composite scores are calculated as the sum of the four domain scores (range 4–24). Due to rounding, composite means are not exactly equal to the sum of domain means.

**Table 2:** Subspecialty-level composite Likert scores for open-ended responses.

Subspecialty Section	n	ChatGPT	Gemini	DeepSeek	*p*-Value
**Laboratory Medicine** *(Cases 1–32)*	32	22.7 ± 1.9	21.4 ± 2.4	20.9 ± 2.5	<0.001
**Histopathology** *(Cases 33–58)*	26	22.3 ± 2.1	20.9 ± 2.5	20.5 ± 2.6	<0.001
**Haematology** *(Cases 59–78)*	20	22.1 ± 2.2	20.6 ± 2.6	20.1 ± 2.7	<0.001
**Microbiology** *(Cases 79–100)*	22	22.8 ± 1.8	21.3 ± 2.3	20.8 ± 2.4	<0.001

Across the three models within each subspecialty, the Friedman test for repeated measures. Composite scores are calculated as the sum of the four domain scores (diagnostic accuracy, interpretation, management, safety), each rated on a six-point Likert scale (1–6). Higher scores indicate better overall performance within that subspecialty.

**Table 3:** Multiple-choice question accuracy by model and subspecialty.

Subspecialty	n	ChatGPT-5.2 *n (%) [95% CI]*	Gemini 3 Pro *n (%) [95% CI]*	DeepSeek-V3.2 *n (%) [95% CI]*
**Laboratory Medicine** *(Cases 1–32)*	160	156 (97.5) [93.7–99.3]	152 (95.0) [90.4–97.8]	151 (94.4) [89.6–97.4]
**Histopathology** *(Cases 33–58)*	130	125 (96.2) [91.3–98.7]	123 (94.6) [89.2–97.8]	121 (93.1) [87.3–96.8]
**Haematology** *(Cases 59–78)*	100	96 (96.0) [90.1–98.9]	96 (96.0) [90.1–98.9]	94 (94.0) [87.4–97.8]
**Microbiology** *(Cases 79–100)*	110	103 (93.6) [87.3–97.4]	104 (94.5) [88.5–98.0]	104 (94.5) [88.5–98.0]
**Overall**	500	480 (96.0) [93.9–97.5]	475 (95.0) [92.7–96.7]	470 (94.0) [91.5–95.9]

Item-level comparison across the three models within each subspecialty (and overall); Values are number correct (%), with exact 95% binomial confidence intervals. Subspecialty-level MCQ accuracy is presented descriptively because the individual paired item-level correctness matrix required for valid repeated-measures or cluster-aware comparative inference was not available for reanalysis. No subgroup inferential *p*-values were reconstructed from marginal counts. Accuracy is defined as the selection of the pre-specified correct option on single-best-answer MCQs (four options, A–D). Percentages are calculated as the number of correct responses divided by the total number of MCQs in each subspecialty or overall.

## Data Availability

Permitted reproducibility materials, including the standardized prompt framework, scoring rubric, model metadata, reference-standard framework, MCQ methodology, and the case index and benchmark structure, are provided in the Supplementary Materials. The source clinical vignettes, original textbook questions, textbook answers and discussions, and other copyright-protected source content from the cited textbook are not reproduced or redistributed in the article or Supplementary Materials. Readers with lawful access to the cited source can identify the underlying cases using the provided case identifiers and section information.

## Supplementary Materials

The following supporting information can be downloaded at: https://www.mdpi.com/article/10.3390/diagnostics16183030/s1, Table S1. Model Metadata and Reproducibility Conditions; Table S2. Open-Ended Response Scoring Rubric; Table S3. Case-Level Performance, Safety Flags, and MCQ Scores for the 100 Benchmark Cases; Table S4. Examples of Reference-Standard Adjudication; Supplementary Methods S1. Development, Internal Review, and Scoring of Investigator-Developed Multiple-Choice Questions; Supplementary Methods S2. Standardized Prompt Template and Interaction Protocol.
