# Supplementary material for: Laboratory Medicine Decision Support—Beyond Exam Passing: A Blinded 100-Case Text-Based Benchmark of Diagnostic Accuracy, Management Quality, and Safety for ChatGPT, Gemini, and DeepSeek—LLM Decision Support in Laboratory Medicine
Source: Diagnostics (Basel). 2026 Sep 18;16(18):3030. doi: 10.3390/diagnostics16183030 (PMC13604957; doi:10.3390/diagnostics16183030)
Supplement: Supplementary file 1 [file diagnostics-16-03030-s001.zip › Supplementary_Methods_S1_MCQ_Development_and_Scoring.pdf]

Supplementary Methods S1. Development, Internal Review, and Scoring of Investigator-Developed Multiple-Choice Questions

This supplementary section describes the development, internal review, administration, and scoring framework for the investigator-developed multiple-choice questions (MCQs) used as a complementary benchmark in the study. It is intended to improve methodological transparency without reproducing copyrighted textbook vignette text or original textbook questions.

S1.1. MCQ Development Framework

Five new MCQs were developed for each of the 100 source cases, yielding 500 MCQs in total. The items were created by two board-certified specialists in clinical pathology and laboratory medicine, each with more than 10 years of professional experience. The MCQs were designed to test clinically relevant decisions arising from the same case material used for the open-ended component.

Each item followed a single-best-answer format with four labeled response options (A-D). Item construction emphasized one clearly preferred answer and avoided multiple-response formats. The targeted task types included identification of the most likely diagnosis, selection of the most appropriate subsequent investigation, recognition or interpretation of a key laboratory/pathology pattern, selection of an appropriate management step, and avoidance of unsafe or inappropriate management.

S1.2. Internal Review and Answer-Key Formation

Draft items, response options, and answer keys were jointly reviewed by the two specialists. Review focused on clinical plausibility, internal consistency with the source case, clarity of the stem and options, appropriateness of the single-best-answer structure, and correctness of the keyed response. Items and answer keys were refined until consensus was reached. The consensus answer key used for scoring was finalized before the MCQs were subsequently presented to the evaluated LLMs.

No independent external specialist panel and no formal psychometric validation procedure were used. Accordingly, the MCQ component should be interpreted as an investigator-developed complementary benchmark rather than as an externally validated examination instrument.

S1.3. Administration to the LLMs

For the MCQ component, the case material was presented in abbreviated form together with the five investigator-developed MCQs. Each MCQ contained four labeled options (A-D). Models were instructed to select exactly one option for each item and to provide a brief justification. Selection of the option letter, rather than the quality of the accompanying justification, determined MCQ correctness.

S1.4. Scoring and Derived MCQ Outcomes

For each item, a response was coded as correct when the model-selected option matched the prespecified consensus answer key and incorrect otherwise. For each case and model, an MCQ score from 0 to 5 was calculated as the number of correctly answered items. Study-level summaries included overall MCQ accuracy and accuracy stratified by textbook section. The MCQ analysis was analytically distinct from the expert-adjudicated reference templates used to score open-ended responses.

S1.5. Domains Represented in the MCQ Set

MCQ task domain	Operational purpose	Typical item focus
Most likely diagnosis	Assess whether the model identifies the principal diagnostic conclusion supported by the vignette.	Selection of the best-supported diagnosis among four alternatives.
Next investigation	Assess prioritization of the most appropriate subsequent diagnostic step.	Selection of a confirmatory or discriminating investigation.
Laboratory/pathology interpretation	Assess recognition of a clinically meaningful pattern in laboratory or pathology information.	Interpretation of a biochemical, hematologic, microbiologic, or pathology pattern.
Management decision	Assess selection of an appropriate management or follow-up action.	Selection of the most appropriate management step under the vignette conditions.
Safety / inappropriate action avoidance	Assess recognition of an action that would be unsafe, contraindicated, or clinically inappropriate.	Identification or avoidance of a potentially harmful management choice.

S1.6. Copyright-Safe Illustrative Item Shells

The following templates illustrate the structure of the investigator-developed items. They are generic item shells, not administered study items, and contain no reproduced textbook vignette text, textbook question, or answer.

Diagnostic item shell

Based on the clinical and laboratory information provided, which of the following is the most likely diagnosis?
A. [Diagnosis A]
B. [Diagnosis B]

C. [Diagnosis C]
D. [Diagnosis D]

Investigation item shell

Which of the following is the most appropriate next investigation to clarify the principal diagnostic question?
A. [Investigation A]
B. [Investigation B]
C. [Investigation C]
D. [Investigation D]

Interpretation item shell

Which option best explains the key laboratory/pathology pattern described in the case?
A. [Interpretation A]
B. [Interpretation B]
C. [Interpretation C]
D. [Interpretation D]

Management item shell

Which of the following is the most appropriate next management step under the conditions described?
A. [Management A]
B. [Management B]
C. [Management C]
D. [Management D]

Safety item shell

Which of the following actions would be least appropriate or potentially unsafe in this clinical context?
A. [Action A]
B. [Action B]
C. [Action C]
D. [Action D]

S1.7. Validation Status and Interpretation

The MCQ set underwent internal specialist consensus review but not independent external validation. Therefore, high MCQ accuracy should be interpreted as performance on this prespecified investigator-developed benchmark. The absence of external item validation is acknowledged as a methodological limitation because the same study specialists contributed to MCQ development and to construction of the open-ended reference framework, creating a potential source of alignment bias.

Future studies should consider independent item development or external answer-key verification and, where the MCQ instrument is intended to function as a formal assessment tool, psychometric evaluation of item difficulty, discrimination, and reliability.

S1.8. Reproducibility and Copyright Note

To preserve copyright compliance, the supplementary materials do not reproduce the source textbook vignettes, original textbook questions, answers, discussions, figures, or tables. The item-development framework, scoring rules, and generic item shells are provided to permit methodological replication while respecting the rights associated with the source material.
