# Supplementary material for: Laboratory Medicine Decision Support—Beyond Exam Passing: A Blinded 100-Case Text-Based Benchmark of Diagnostic Accuracy, Management Quality, and Safety for ChatGPT, Gemini, and DeepSeek—LLM Decision Support in Laboratory Medicine
Source: Diagnostics (Basel). 2026 Sep 18;16(18):3030. doi: 10.3390/diagnostics16183030 (PMC13604957; doi:10.3390/diagnostics16183030)
Supplement: Supplementary file 1 [file diagnostics-16-03030-s001.zip › Supplementary_Methods_S2_Standardized_Prompt_and_Interaction_Protocol.pdf]

Supplementary Methods S2. Standardized Prompt Template and Interaction Protocol

This supplement documents the standardized user-facing prompt structure and interaction sequence used in the benchmark. To comply with copyright restrictions, we represent source textbook vignette text and original textbook questions with placeholders rather than reproducing them verbatim. The templates below therefore preserve the documented experimental structure without redistributing protected content.

S2.1. Open-Ended Prompt Structure

Prompt element	Copyright-safe representation
**Case identifier**	[CASE ID / SOURCE CASE NUMBER]
**Textbook section**	[LABORATORY MEDICINE / HISTOPATHOLOGY / HAEMATOLOGY / MICROBIOLOGY]
**Clinical vignette**	[SOURCE CLINICAL VIGNETTE — COPYRIGHTED TEXT NOT REPRODUCED]
**Source questions**	[CASE-SPECIFIC SOURCE OPEN-ENDED QUESTIONS — NOT REPRODUCED]
**Instruction block**	Answer each numbered question in order. Provide concise, guideline-concordant reasoning suitable for a senior resident. If information is insufficient or uncertainty remains, state this explicitly rather than guessing.

Important note: The wording above is a copyright-safe representation of the prompt structure documented in the Methods. Where an exact archived investigator prompt exists, that archived wording should be treated as the authoritative study record.

S2.2. MCQ Prompt Structure

Prompt element	Copyright-safe representation
**Case context**	[ABBREVIATED CASE CONTEXT DERIVED FROM THE SAME SOURCE CASE — SOURCE TEXT NOT REPRODUCED]
**MCQ set**	[FIVE INVESTIGATOR-DEVELOPED SINGLE-BEST-ANSWER MCQs; FOUR OPTIONS, A–D]
**Instruction block**	Select exactly one option (A, B, C, or D) for each question and provide a brief justification in one or two sentences. Do not omit an item or select more than one option.

S2.3. Interaction Sequence

Step	Procedure	Purpose / control
1	Initiate a new conversation for the case.	Minimize cross-case contextual carryover.
2	Present the standardized case header, source vignette, and source open-ended questions.	Provide the same case content and question structure across evaluated models.
3	Apply the standardized open-ended instruction block.	Standardize requested response format and handling of uncertainty.
4	Save the completed open-ended response before presenting MCQs.	Prevent the later MCQ response from influencing blinded open-ended scoring.
5	Within the same case conversation, present the abbreviated case context and five investigator-developed MCQs.	Obtain the complementary single-best-answer assessment for the same case.
6	Require one option (A–D) per MCQ plus a brief justification.	Standardize MCQ response format; correctness is based on the selected option letter.
7	Record the interaction timestamp, response latency, and total response length.	Preserve interaction-level metadata for reproducibility and descriptive analysis.
8	Export responses and remove model-identifying/platform-specific formatting before blinded scoring.	Maintain rater blinding.

S2.4. Operator and Platform Controls

Control	Documented study condition
**Prompt entry**	A clinician researcher trained in laboratory medicine entered prompts manually.
**Model-order handling**	The order in which models received each case was randomized using a computer-generated list, as documented in the Methods.
**Language**	All interactions were conducted in English.
**Browsing / external resources**	Browsing or tool use was disabled where an explicit user control was available; when complete deactivation could not be verified, the prompt instructed the model not to search the web or access external resources.
**Generation parameters**	No undocumented numerical temperature, top-p, seed, or backend decoding setting is retrospectively assigned in this supplement.
**Conversation state**	We initiated a new conversation for each case to minimize carryover; this does not establish complete proprietary platform-level statelessness.

S2.5. Reproducibility and Copyright Boundary

The reproducibility materials intentionally separate the experimental procedure from protected source content. Case identifiers, textbook section, interaction sequence, instruction structure, scoring framework, model metadata, and investigator-developed methodological materials can be reported. Full source clinical vignettes, original textbook questions, textbook answers/discussions, figures, tables, or substantial extracts are not reproduced or redistributed. Readers with lawful access to the cited textbook can map the study-facing case identifiers to the source cases using Supplementary Table S3.

This supplement does not claim access to provider-side hidden system instructions, backend routing, proprietary checkpoint identifiers, or other non-user-controllable platform settings.
