# Supplementary material for: Laboratory Medicine Decision Support—Beyond Exam Passing: A Blinded 100-Case Text-Based Benchmark of Diagnostic Accuracy, Management Quality, and Safety for ChatGPT, Gemini, and DeepSeek—LLM Decision Support in Laboratory Medicine
Source: Diagnostics (Basel). 2026 Sep 18;16(18):3030. doi: 10.3390/diagnostics16183030 (PMC13604957; doi:10.3390/diagnostics16183030)
Supplement: Supplementary file 1 [file diagnostics-16-03030-s001.zip › Supplementary_Table_S1_Model_Metadata_and_Reproducibility.pdf]

Supplementary Table S1. Model Metadata and Reproducibility Conditions

This table reports only user-facing and study-recorded information that can be supported by the available manuscript and revision archive. Proprietary backend identifiers, hidden system instructions, and undocumented generation parameters were not retrospectively inferred.

Variable	ChatGPT-5.2	Gemini 3 Pro	DeepSeek-V3.2
**Provider**	OpenAI	Google DeepMind	DeepSeek AI
**User-visible model label**	ChatGPT-5.2 Thinking	Gemini 3 Pro	DeepSeek-V3.2
**Access route**	Official ChatGPT consumer web interface	Official Gemini consumer web interface	Official DeepSeek consumer web interface
**Input modality**	English text only	English text only	English text only
**Exact access date(s)**	Not available in the current revision archive; to be transcribed only from the contemporaneously recorded interaction timestamps.	Not available in the current revision archive; to be transcribed only from the contemporaneously recorded interaction timestamps.	Not available in the current revision archive; to be transcribed only from the contemporaneously recorded interaction timestamps.
**Account / subscription tier**	Not documented in the available study archive; not retrospectively inferred.	Not documented in the available study archive; not retrospectively inferred.	Not documented in the available study archive; not retrospectively inferred.
**Conversation handling**	A new conversation was initiated for each case to minimize cross-case contextual carryover.	A new conversation was initiated for each case to minimize cross-case contextual carryover.	A new conversation was initiated for each case to minimize cross-case contextual carryover.
**Prompt delivery**	Manual entry by a clinician researcher using the standardized protocol.	Manual entry by a clinician researcher using the standardized protocol.	Manual entry by a clinician researcher using the standardized protocol.
**Reasoning / thinking mode**	The user-visible model label included “Thinking”; no additional backend reasoning configuration was independently accessible.	No separate user-configurable reasoning setting was documented in the available study archive.	No separate user-configurable reasoning setting was documented in the available study archive.
**Temperature**	No reproducible user-configured value documented; the previous statement that temperature was set to 0 is removed.	No reproducible user-configured value documented; not retrospectively inferred.	No reproducible user-configured value documented; not retrospectively inferred.
**Top-p / other sampling parameters**	Not documented as user-configured in the available study archive; not retrospectively inferred.	Not documented as user-configured in the available study archive; not retrospectively inferred.	Not documented as user-configured in the available study archive; not retrospectively inferred.
**Seed**	Not documented / not retrospectively inferred.	Not documented / not retrospectively inferred.	Not documented / not retrospectively inferred.
**Safety controls**	Provider-default safety controls retained.	Provider-default safety controls retained.	Provider-default safety controls retained.
**Browsing / web search**	Disabled where an explicit interface control was available; otherwise the study prompt explicitly prohibited external web search or external-resource access.	Disabled where an explicit interface control was available; otherwise the study prompt explicitly prohibited external web search or external-resource access.	Disabled where an explicit interface control was available; otherwise the study prompt explicitly prohibited external web search or external-resource access.
**External tools / files / retrieval augmentation**	No investigator-supplied tools, external files, plug-ins, or retrieval-augmented sources were used in the intended study protocol.	No investigator-supplied tools, external files, plug-ins, or retrieval-augmented sources were used in the intended study protocol.	No investigator-supplied tools, external files, plug-ins, or retrieval-augmented sources were used in the intended study protocol.
**Memory / personalization**	Fresh conversations minimized conversation-level carryover; platform-level memory/personalization state was not independently verifiable from the available archive.	Fresh conversations minimized conversation-level carryover; platform-level memory/personalization state was not independently verifiable from the available archive.	Fresh conversations minimized conversation-level carryover; platform-level memory/personalization state was not independently verifiable from the available archive.
**Custom instructions / hidden system instructions**	Researcher-supplied study instructions were standardized. Provider-side hidden system instructions were inaccessible.	Researcher-supplied study instructions were standardized. Provider-side hidden system instructions were inaccessible.	Researcher-supplied study instructions were standardized. Provider-side hidden system instructions were inaccessible.
**Backend build / checkpoint / routing**	Not exposed to the investigators; not inferred.	Not exposed to the investigators; not inferred.	Not exposed to the investigators; not inferred.
**Interaction metadata recorded**	Timestamp, response latency, and total response length were recorded for each interaction.	Timestamp, response latency, and total response length were recorded for each interaction.	Timestamp, response latency, and total response length were recorded for each interaction.

Notes. “Not documented” means that the item could not be verified from the currently available study/revision archive and was therefore not reconstructed. Exact access dates should be entered only after checking the contemporaneous timestamp logs. Use of a fresh conversation reduces, but does not prove the absence of, proprietary platform-level memory, personalization, routing, or hidden context. The table describes the user-facing experimental conditions and does not claim reproducibility of the providers’ inaccessible backend state.
