# Supplementary material for: Laboratory Medicine Decision Support—Beyond Exam Passing: A Blinded 100-Case Text-Based Benchmark of Diagnostic Accuracy, Management Quality, and Safety for ChatGPT, Gemini, and DeepSeek—LLM Decision Support in Laboratory Medicine
Source: Diagnostics (Basel). 2026 Sep 18;16(18):3030. doi: 10.3390/diagnostics16183030 (PMC13604957; doi:10.3390/diagnostics16183030)
Supplement: Supplementary file 1 [file diagnostics-16-03030-s001.zip › Supplementary_Table_S3.pdf]

Table S3. Case-Level Performance, Safety Flags, and MCQ Scores for the 100 Benchmark Cases.

Case No	Section	ChatGPT score	ChatGPT Safety Flag	ChatGPT MCQ (0-5)	Gemini Score	Gemini Safety Flag	Gemini MCQ (0-5)	DeepSeek Score	DeepSeek Safety Flag	DeepSeek MCQ (0-5)
1	Laboratory Medicine	21	No	5	22	No	5	24	No	5
2	Laboratory Medicine	23	No	5	22	No	5	21	No	5
3	Laboratory Medicine	24	No	5	19	No	4	23	No	5
4	Laboratory Medicine	24	No	5	20	No	5	17	No	4
5	Laboratory Medicine	19	No	4	19	No	4	22	No	5
6	Laboratory Medicine	24	No	5	21	No	5	24	No	5
7	Laboratory Medicine	24	No	5	24	No	5	16	Yes	3
8	Laboratory Medicine	24	No	5	20	No	5	19	No	4
9	Laboratory Medicine	24	No	5	20	No	5	23	No	5
10	Laboratory Medicine	23	No	5	16	Yes	3	23	No	5
11	Laboratory Medicine	24	No	5	24	No	5	20	No	5
12	Laboratory Medicine	24	No	5	18	No	4	23	No	5
13	Laboratory Medicine	23	No	5	19	No	5	19	No	5
14	Laboratory Medicine	24	No	5	24	No	5	23	No	5
15	Laboratory Medicine	20	No	4	24	No	5	24	No	5
16	Laboratory Medicine	24	No	5	19	No	5	24	No	5
17	Laboratory Medicine	19	No	4	24	No	5	17	No	4
18	Laboratory Medicine	23	No	5	22	No	5	18	No	4
19	Laboratory Medicine	23	No	5	23	No	5	17	No	4
20	Laboratory Medicine	17	Yes	4	23	No	5	20	No	5
21	Laboratory Medicine	21	No	5	24	No	5	18	No	4
22	Laboratory Medicine	22	No	5	21	No	5	24	No	5
23	Laboratory Medicine	24	No	5	18	No	4	22	No	5
24	Laboratory Medicine	22	No	5	22	No	5	24	No	5
25	Laboratory Medicine	24	No	5	24	No	5	21	No	5
26	Laboratory Medicine	21	No	5	21	No	5	22	No	5
27	Laboratory Medicine	24	No	5	24	No	5	21	No	5
28	Laboratory Medicine	24	No	5	24	No	5	22	No	5
29	Laboratory Medicine	24	No	5	18	No	4	22	No	5
30	Laboratory Medicine	24	No	5	24	No	5	20	No	5
31	Laboratory Medicine	24	No	5	24	No	5	19	No	5
32	Laboratory Medicine	21	No	5	18	No	4	17	No	4
33	Histopathology	20	No	4	21	No	5	24	No	5

34	Histopathology	18	Yes	4	24	No	5	24	No	5
35	Histopathology	24	No	5	24	No	5	21	No	5
36	Histopathology	24	No	5	21	No	5	17	No	4
37	Histopathology	24	No	5	21	No	5	22	No	5
38	Histopathology	24	No	5	24	No	5	23	No	5
39	Histopathology	24	No	5	21	No	5	21	No	5
40	Histopathology	23	No	5	23	No	5	19	No	4
41	Histopathology	24	No	5	24	No	5	19	No	4
42	Histopathology	24	No	5	21	No	5	21	No	5
43	Histopathology	20	No	4	23	No	5	20	No	5
44	Histopathology	24	No	5	15	Yes	3	18	No	4
45	Histopathology	18	No	4	24	No	5	21	No	5
46	Histopathology	22	No	5	21	No	5	16	Yes	4
47	Histopathology	24	No	5	20	No	5	15	Yes	3
48	Histopathology	20	No	5	21	No	5	19	No	5
49	Histopathology	21	No	5	19	No	4	23	No	5
50	Histopathology	24	No	5	18	No	4	20	No	5
51	Histopathology	22	No	5	16	Yes	4	23	No	5
52	Histopathology	23	No	5	20	No	5	24	No	5
53	Histopathology	22	No	5	18	No	4	22	No	5
54	Histopathology	23	No	5	19	No	5	20	No	5
55	Histopathology	23	No	5	24	No	5	17	No	4
56	Histopathology	23	No	5	21	No	5	24	No	5
57	Histopathology	18	No	4	18	No	4	18	No	4
58	Histopathology	24	No	5	22	No	5	22	No	5
59	Haematology	24	No	5	24	No	5	23	No	5
60	Haematology	24	No	5	23	No	5	20	No	5
61	Haematology	24	No	5	24	No	5	24	No	5
62	Haematology	19	No	4	22	No	5	20	No	5
63	Haematology	22	No	5	23	No	5	16	Yes	4
64	Haematology	24	No	5	16	Yes	4	20	No	5
65	Haematology	22	No	5	24	No	5	20	No	5
66	Haematology	24	No	5	21	No	5	24	No	5
67	Haematology	18	No	4	21	No	5	24	No	5
68	Haematology	24	No	5	18	No	5	17	No	4

69	Haematology	24	No	5	17	No	4	19	No	5
70	Haematology	18	No	4	19	No	5	16	No	4
71	Haematology	22	No	5	22	No	5	23	No	5
72	Haematology	19	No	4	17	No	4	20	No	5
73	Haematology	21	No	5	23	No	5	18	No	4
74	Haematology	24	No	5	19	No	5	19	No	5
75	Haematology	20	No	5	22	No	5	17	No	4
76	Haematology	21	No	5	19	No	5	18	No	4
77	Haematology	24	No	5	17	No	4	24	No	5
78	Haematology	24	No	5	21	No	5	20	No	5
79	Microbiology	23	No	4	22	No	5	19	No	4
80	Microbiology	20	No	4	17	No	3	18	No	4
81	Microbiology	24	No	5	22	No	5	21	No	5
82	Microbiology	23	No	5	24	No	5	20	No	4
83	Microbiology	24	No	5	18	No	4	22	No	5
84	Microbiology	24	No	5	24	No	5	24	No	5
85	Microbiology	22	No	4	20	No	5	24	No	5
86	Microbiology	23	No	5	24	No	5	23	No	5
87	Microbiology	24	No	5	20	No	5	20	No	5
88	Microbiology	24	No	5	21	No	5	21	No	5
89	Microbiology	18	No	4	23	No	5	24	No	5
90	Microbiology	19	No	4	24	No	5	17	No	4
91	Microbiology	24	No	5	22	No	5	18	No	4
92	Microbiology	24	No	5	18	No	4	23	No	5
93	Microbiology	24	No	5	17	No	4	20	No	5
94	Microbiology	24	No	5	23	No	5	22	No	5
95	Microbiology	23	No	5	23	No	5	21	No	5
96	Microbiology	21	No	4	19	No	4	24	No	5
97	Microbiology	24	No	5	23	No	5	15	**Yes**	4
98	Microbiology	24	No	5	23	No	5	20	No	5
99	Microbiology	22	No	4	20	No	5	20	No	5
100	Microbiology	24	No	5	22	No	5	22	No	5
