# Supplementary material for: Laboratory Medicine Decision Support—Beyond Exam Passing: A Blinded 100-Case Text-Based Benchmark of Diagnostic Accuracy, Management Quality, and Safety for ChatGPT, Gemini, and DeepSeek—LLM Decision Support in Laboratory Medicine
Source: Diagnostics (Basel). 2026 Sep 18;16(18):3030. doi: 10.3390/diagnostics16183030 (PMC13604957; doi:10.3390/diagnostics16183030)
Supplement: Supplementary file 1 [file diagnostics-16-03030-s001.zip › Supplementary_Table_S4_Reference_Standard_Adjudication.pdf]

Supplementary Table S4. Examples of Reference-Standard Adjudication

Purpose. This table documents the prespecified adjudication logic used to construct the case-specific reference templates from the textbook-derived pedagogical answers, specialist review, and contemporary major guidelines or consensus recommendations where directly applicable. The textbook answer served as the starting pedagogical reference rather than an immutable gold standard.

Adjudication scenario	Textbook-derived starting point	Specialist / guideline review	Final reference-standard rule	Scoring implication
1. Textbook and contemporary guidance concordant	The textbook answer identifies the expected diagnosis, interpretation, investigation, and/or management element.	The two specialists confirm that the element remains clinically appropriate and is consistent with directly applicable contemporary guidance.	The shared element is retained in the expert-adjudicated reference template.	Responses containing the essential element are considered concordant; equivalent wording is accepted.
2. A newer directly applicable guideline materially differs from the textbook	The textbook provides the pedagogical answer available at publication.	A more recent major guideline or consensus recommendation is identified and judged by both specialists to be directly applicable to the case.	The current guideline-concordant recommendation takes precedence for the affected element. The textbook is not treated as an immutable gold standard.	A model is not penalized for following the current, directly applicable guideline rather than an outdated textbook formulation.
3. More than one management or investigation pathway is clinically acceptable	The textbook may present one preferred pathway.	Specialist review identifies additional guideline-concordant alternatives that would be considered acceptable in routine practice.	The permissible response range is broadened to include all clinically acceptable, guideline-concordant options.	Scoring is based on clinical acceptability and completeness, not exact textual agreement with a single textbook pathway.
4. No directly applicable contemporary guideline exists	The textbook provides the primary pedagogical framework.	The specialists review the case using accepted laboratory-medicine and clinical-pathology principles and determine whether the textbook formulation remains reasonable.	The textbook-derived element is retained when no stronger, directly applicable contemporary recommendation supersedes it.	The reference template remains anchored to the pedagogical source, with expert review defining acceptable equivalent responses.
5. Terminology differs but the clinical meaning is equivalent	The textbook uses a specific diagnostic or interpretative term.	The specialists determine that synonymous or updated nomenclature represents the same clinically relevant concept.	Clinically equivalent terminology is accepted in the reference template.	Models are not penalized solely for using accepted contemporary terminology or a clinically equivalent synonym.
6. Diagnostic uncertainty or a defensible differential remains	The textbook may emphasize a single leading diagnosis.	Specialist review determines whether the available vignette supports only one diagnosis or whether a narrow differential is clinically defensible.	The reference template specifies the required leading diagnosis and, where appropriate, permissible differential diagnoses and qualifying uncertainty.	A response may receive partial or full credit depending on whether the leading diagnosis and clinically appropriate uncertainty are represented.
7. Safety-sensitive management recommendation	The textbook provides an investigation or management recommendation.	The specialists assess compatibility with current safety standards and major guidance where applicable.	Only recommendations judged clinically acceptable and non-harmful are included in the permissible reference range.	Clearly unsafe or guideline-discordant advice can reduce the management/safety score and may trigger the binary high-threshold safety flag.
8. Reference-standard construction versus output-scoring adjudication	The reference template is constructed before model-output scoring from textbook-derived elements, specialist review, and applicable guidance.	This process is kept conceptually separate from later disagreements between blinded raters when scoring model outputs.	The final reference template is used as the comparison framework for scoring. A third expert is invoked only for unresolved scoring disagreements if documented, not retroactively for reference-standard formation.	Prevents conflation of reference-standard construction with post hoc model-output adjudication.

Important transparency note. The study files currently available do not contain a prospectively maintained case-level log identifying specific textbook-versus-guideline discordance events. Accordingly, no retrospective or hypothetical case-specific adjudication event has been presented as if it occurred during the original study. This table therefore reports the documented adjudication framework and permissible decision rules used for reference-template construction. If contemporaneous case-level adjudication notes are recovered, those records can be appended as case-specific examples without altering the study results.

Abbreviations: LLM, large language model. “Guideline” includes a contemporary major clinical guideline or consensus recommendation judged by the specialists to be directly applicable to the clinical scenario.
